# Design of twin delayed deep deterministic policy gradient RL based adaptive controller for DC motor speed regulation considering uncertainties

**DOI:** 10.1038/s41598-025-33644-w

**Published:** 2026-01-09

**Authors:** P. Gaur, V. P. Singh, T. Varshney, P. Sanjeevikumar, A. Mathur

**Affiliations:** 1 Department of Electrical Engineering, Malaviya National Institute of Technology, Jaipur, 302017 India; 2 Department of Electrical Electronics and Communication Engineering, Sharda University, Greater Noida, UP 201310 India; 3 Department of Electrical Engineering, IT and Cybernetics, University of South-Eastern Norway, Porsgrunn, Norway; 4 Department of Electrical Engineering, Malaviya National Institute of Technology, Jaipur, 302017 India

**Keywords:** Reinforcement learning, TD3 Algorithm, FOPDT model, DC motor, Speed regulation, Convergence curve, Setpoint filter, Electrical and electronic engineering, Mathematics and computing

## Abstract

Reinforcement learning offers efficient solutions for optimizing complex decision-making tasks through continuous state-action-reward cycle with real-time adaptability. This work presents twin delayed deep deterministic (TD3) policy gradient RL based adaptive speed controller for the DC motor model while considering the impact of various uncertainties from dynamic environment into account. Various benchmark controller techniques are also utilized for similar objective in order to perform comparative analysis. Responses of each controller are plotted for both constant and variable desired speeds to evaluate their efficacy, robustness, and adaptability to uncertainties. Values of various types of error indices, including integral of squared error (ISE), integral of time-weighted absolute error (ITAE), integral of absolute error (IAE), integral of time-weighted squared error (ITSE), and their respective time-weighted variants are calculated, and tabulated for each type of speed controller for both test cases. Error indices analysis is also utilized to compare, and evaluate each controller’s tracking precision and error minimization qualities in dynamic operating conditions for efficient speed regulation for the DC motor.

## Introduction

Reinforcement learning (RL) is a type of machine learning technique where an algorithm learns to make sequential decisions by optimizing its actions in order to maximize a cumulative reward^1,2^. RL has caught attention of researchers of various domains due to it’s capabilities of improving efficiency, save energy, and make more accurate predictions in various industrial application^3,4^. It has proven invaluable potential in real-time decision-making and optimization tasks. Moreover, RL is transforming healthcare sector, where it aids in medical image analysis, radiology reports, real-time health monitoring, personalized treatment plans, and drug discovery^5,6^. The electrical vehicle domain is also witnessing technological advancement due to implementation of RL in load forecasting in charging station, improved battery life, vehicle-to-grid (V2G), and real-time controls^7–9^. RL assisted automated trading and portfolio management are upgrading financial sector^10,11^. The power sector is also going through noteworthy up-gradation due to integration of RL techniques in load-generation balance, optimal resource allocation, and unit commitment tasks^12–14^. Additionally, RL is widely applied in areas such as military^15^, cyber security^16^, aviation^17,18^, cryptocurrency^19,20^, smart agriculture^21,22^, etc.

RL has evolved from fundamental concepts to advance algorithms over the years in order to address complex decision-making problems. The concept of optimality, which laid the framework for Markov decision processes (MDPs) and dynamic programming, was introduced by Bellman^23^. Watkins developed Q-learning^24^, a model-free approach for learning state-action values. Mnih et al.^25^ revolutionized RL with DQN, enabling high-dimensional state space handling by fusing Q-learning and deep neural networks. To extend RL to continuous actions, DPG^26^ was introduced, followed by DDPG^27^, which used an actor-critic framework for improved stability. Twin delayed deep deterministic (TD3) policy gradient algorithm^28^ refined DDPG by addressing overestimation bias and enhancing exploration, making it more efficient for continuous control tasks.

Following the exploration of RL techniques in control systems engineering in various domains, it is essential to understand the fundamental dynamics and control challenges associated with DC motors. DC motors remain a widely used machine in diverse industrial fields such as aviation^29^, electrical vehicle^30^, robotics^31^, irrigation^32,33^, etc. The performance of DC motor is impacted by various external factors such as presence of moisture, dirt, and dust^34^. Performance variation is also observed for the DC motor as the operating temperature changes^35^. Both unbalanced power supply, and failed ventilation system cause degradation in efficacy of the DC motor^34^. The speed control is crucial for ensuring precise performance, energy efficiency, and stability under dynamic operating conditions^36,37^.

Various types of controller designing techniques have been utilized in past research works e.g. AMIGO method^38^, Cohen-Coon (CC) method^39^, Ho’s method^40^, and Zigler-Nichols (ZN) method^41^. These controller techniques^38–41^ may get stuck in sub-optimal solutions, exhibit limited adaptability to disturbances, and struggle with uncertainties in environments. Metaheuristic algorithms like moth flame optimization (MFO)^42^, cuckoo optimization algorithm (COA)^43^, grey wolf optimization (GWO)^44^, whale optimization algorithm (WOA)^45^, and ant colony optimization (ACO)^46^ algorithm are also implemented for designing controller in order to address various industrial objectives. Still such controllers algorithms^42–46^ suffer from computational complexity, slow convergence speed, and performance degradation due to uncertainties in environments.

As the efficiency of the DC motor system is influenced by a wide range of factors^34,35^, need for efficient controller designing is highly desirable, which is adaptive in nature and performs efficiently while considering various uncertainties in the system environment. Although RL is widely applied for various real life applications by reseachers^5–13,15–22^, yet its application in the DC motor speed regulation application remains relatively underexplored. This research gap highlights the need for further investigation into how RL can be utilized for DC motor system to achieve speed regulation in efficient manner while considering various type of uncertainties.

This article proposed TD3 RL based adaptive speed controller in order to achieve efficient speed regulation for DC motor system while considering various uncertainties from environment. Transfer function (TF) based DC motor model is utilized in this work to design and implement the TD3 RL for similar objective. Further, that DC motor’s TF is approximated into first-order plus dead time (FOPDT) model to calculate gains for various benchmark controllers to perform comparative analysis against the proposed adaptive speed controller. Responses of all designed controllers are plotted for both constant and variable desired speeds to test each controller’s adaptability, transition performance, and stability in dynamic operating conditions. Effectiveness and robustness of each controller are evaluated by calculating key error indices. Later, all these error indices are tabulated and compared to evaluate performance of each controller among themselves.

This research work’s subsequent sections are organized as follows: Section “TD3 policy gradient algorithm” presents the key improvements introduced in TD3 RL compared to DDPG RL, along with the algorithm of TD3 RL. Section “System description” explains TD3 algorithm’s structure implementation for speed regulation for the DC motor model while considering various uncertainties from dynamic environment. This section also explains working and significance of each key components utilized in overall control strategy. In Section “Simulation results and discussion”, the results are plotted for both constant and variable desired speeds. Key error indices are also calculated, and tabulated for comparative analysis in this section. Lastly, Section “Conclusion and future work” encapsulates the article with key outcomes and suggesting pathways for extended research.

## TD3 policy gradient algorithm

TD3 algorithm is one of the recent advancements in RL techniques as it improves DDPG by using methods that increase stability, lower overestimation, and assure robust learning. These updates make TD3’s performance more effective in continuous action spaces by addressing issues such as unstable updates, overfitting, and Q-value estimate errors. TD3 RL is applied by researchers in various industrial applications such as aviation^47^, 5G-communication^48^, robotics^49^, electrical vehicle^50^, hybrid power system^51^, etc. Three major updates in TD3 compared to DDPG are written below.

1. TD3 algorithm utilized twin critic networks in order to estimate Q-value. The lesser of the two values is chosen during target updates. This method will ensure safer policy update and enhance stability of learning throughout training process by reducing the possibility of overestimation and ensures that Q-value estimations stay closer to their actual values.
2. The actor-network undergoes updates less often in TD3 RL compared to the critic network by continuously shifting Q-value parameters in order to avoid instability. This approach ensures more reliable and steady improvements in the actor network over time by delaying policy updates, which avoids repeated adjustments with regard to an unmodified critic.
3. When calculating the Q-value in TD3, noise drawn from a truncated normal distribution is added to the target actions to prevent deterministic policies from overfitting to narrow peaks in value estimations. This lowers the target’s variance and improves robustness, particularly in stochastic situations with possible failure instances.

After the above three key reform, the target values ($y_1$, and $y_2$) for the twin critic networks are computed as per (1).

1 $$\begin{aligned} {\left\{ \begin{array}{ll} y_1 = r_t + \gamma Q'_{\psi _1}(s'_{t+1}, {\tilde{a}}_t) \\ y_2 = r_t + \gamma Q'_{\psi _2}(s'_{t+1}, {\tilde{a}}_t) \end{array}\right. } \end{aligned}$$

where, $Q'_{\psi _1}$ and $Q'_{\psi _2}$ are the target critic networks, $r_t$ is reward, $\gamma$ is discount factor, $s'_{t+1}$ is state, and ${\tilde{a}}_t$ is the noise-added target action. This noise is sampled from a clipped normal distribution and is added to the output of the target actor network as:

2 $$\begin{aligned} {\tilde{a}}_t \leftarrow \eta _{\phi '}(s'_t) + \epsilon , \quad \epsilon \sim \operatorname {clip}\!\left( {\mathcal {N}}(0, {\tilde{\sigma }}), -c, c\right) \end{aligned}$$

Next, to reduce overestimation bias, the minimum of the two calculated target Q-values is utilized as the final target Q-value:

3 $$\begin{aligned} y \leftarrow r_t + \gamma \min _{i=1,2} Q'_{\psi _i}(s'_{t+1}, {\tilde{a}}_t) \end{aligned}$$

The update of the critic networks involves minimizing the mean squared error (MSE) between the computed target value and the current Q-values. Later, the deterministic policy gradient (DPG) update is utilized to modify the actor-network, as shown by:

4 $$\begin{aligned} \nabla _\phi J(\phi ) = N^{-1} \sum \nabla _{a_t} Q_{\psi _1}(s_t, a_t)\Big |_{a_t = \eta _\phi (s_t)} \nabla _\phi \eta _\phi (s_t) \end{aligned}$$

Finally, soft updates are performed for both target-critic networks and target-actor network using Polyak averaging to ensure stable learning dynamics across iterations. The algorithm of TD3 RL is discussed in Algorithm 1.

**Algorithm 1:**
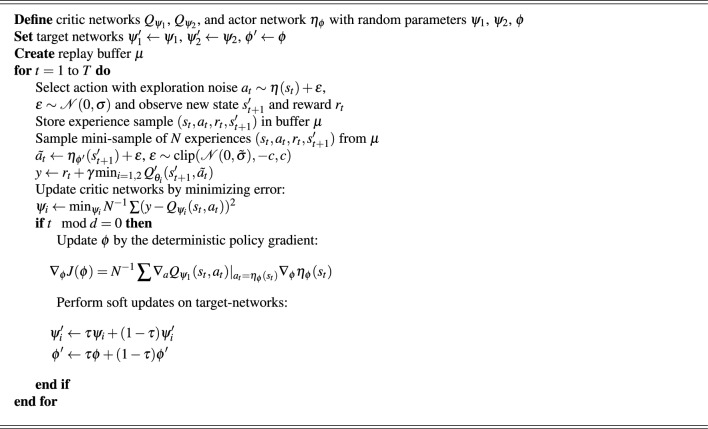
TD3 RL.

Interpretations of various parameters utilized in above TD3 RL algorithm are mention in the Table 1.

**Table 1:** Parameters of TD3 RL algorithm.

S.N.	Parameters	Descriptions
1	$Q_{\psi _1}, Q_{\psi _2}$	Twin critic networks, where $\psi _1$ and $\psi _2$ are their respective weights
2	$\eta _{\phi }$	Actor network, where $\phi$ is its respective weight
3	$\psi _1', \psi _2'$	Weights of target critic networks
4	$\phi '$	Weight of target actor network
5	$\mu$	Replay buffer
6	*T*	Total number of episodes
7	Sample $(s_t, a_t, r_t, s'_{t+1})$	Transition group in which $s_t$, $a_t$, $s'_{t+1}$, and $r_t$ are the state, action, next state, and reward, respectively
8	$\gamma$	Discount factor
9	$\nabla _\varphi J(\varphi )$	A gradient of the actor-network’s loss function
10	$\psi _i'$	Updated value of the critic network’s weight parameter
11	$\phi '$	Adjusted weight parameter’s value for critic-network
12	$\tau$	Adjusted weight parameter’s value for actor-network

## System description

**Fig. 1:**
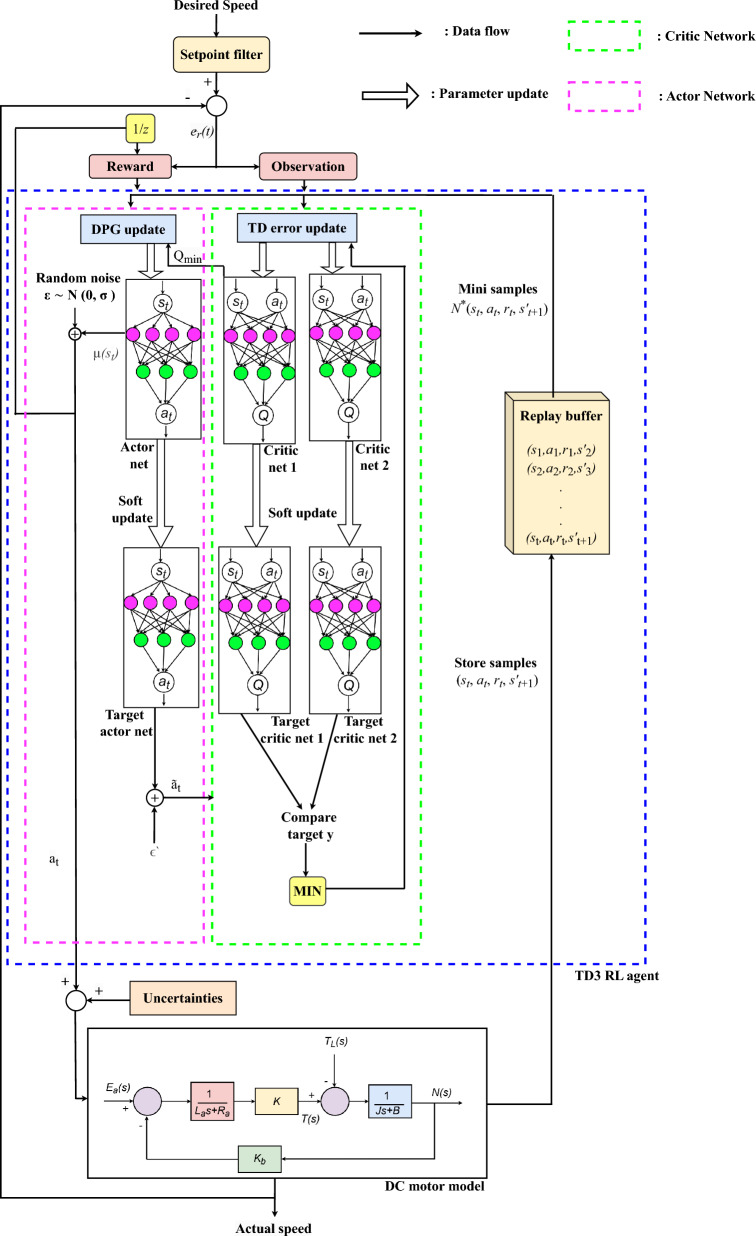
TD3 algorithm’s structure implementation.

This section describes the design and implementation of TD3 RL based adaptive controller for DC motor speed regulation. Overall structural implementation of TD3 RL agent for speed regulation is depicted in Fig. 1. The key components, depicted in Fig. 1, are DC motor, setpoint filter, TD3 RL agent, observation, reward and uncertainties. DC motors are categorized based on methods to supply current to field winding. It can be either self-excited or externally-excited types. In this work, an externally-excited type DC motor is utilized to design of the adaptive speed controller.

The TF of the DC motor^52^ in an open-loop configuration is given as

5 $$\begin{aligned} D_{tf}(s) = \frac{\omega (s)}{V_a(s)} = \frac{K}{(L_a s + R_a)(Js + B) + K_b K} \end{aligned}$$

where $\omega$ and $V_a$ are the angular speed of motor shaft (rad/s) and the armature voltage (V), respectively. Both the representation and the values of various other parameters, as mentioned in (5), are tabulated in Table 2^53–55^. After utilizing values form Table 2, following TF of DC motor is obtained:

6 $$\begin{aligned} D_{tf}(s) = \frac{\omega (s)}{V_a(s)} = \frac{0.015}{0.00108s^2+0.0061s+0.00163} \end{aligned}$$

**Fig. 2:**
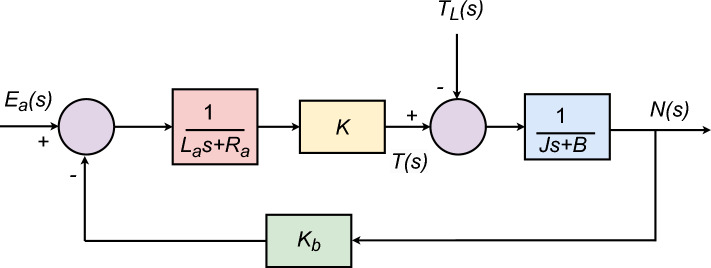
DC motor model.

The block diagram of the DC motor model is illustrated in Fig. 2^52^.

**Table 2:** Parameters of DC motor.

S. N.	Parameter	Symbol	Value
1	Motor torque constant	*K*	0.015 N.m/A
2	Armature inductance	$L_a$	2.7 H
3	Armature resistance	$R_a$	0.4 $\Omega$
4	Inertia torque of motor	*J*	0.0004 kg.m^2^
5	Motor friction constant	*B*	0.0022 N.m.s/rad
6	Electromotive force constant	$K_b$	0.05 V.s/rad

Sharp variation in desired speed demands quick adjustments from DC motor, which might cause sudden excessive stress on mechanical parts. Such events are highly undesirable because they might degrade overall lifetime and result in frequent maintenance of the motor. That’s why a setpoint filter is utilized in overall speed control strategy in order to ensuring smooth transitions, reducing overshoot, and preventing aggressive control actions^56^. First order setpoint filter, which is the applied to reference speed in Fig. 1, is given as:

7 $$\begin{aligned} F(s) = \frac{1}{1+\alpha _fs} \end{aligned}$$

In (7), $\alpha _f$ is coefficient of set-point filter. This time constant helps in minimizing overshoots during speed variations.

TD3 RL agent is utilized in the overall structural implementation of the adaptive speed controller for DC motor system. It’s various hyperparameter and their specifications are tabulated in Table 3. The observation block is utilized in the overall speed control strategy, because it processes the state information ($s_t$) received from the environment and feed that to TD3 RL agent, assisting it to choose right action ($a_t$) for that state. It takes system states such as speed error ($e_r(t)$) as an input in proposed adaptive controller. This block takes $e_r(t)$ and it’s discrete-time integrator as inputs to further process these to TD3 RL agent.

Reward block provides feedback to the TD3 RL agent by evaluating how well $a_t$ contributes to achieving the desired system performance. It also penalizes $e_r(t)$ to encourage accurate tracking to desired speed. It is a critic component which ensures that TD3 agent adapts effectively to various types of uncertainties and dynamic environment. Output from reward block is given as:

8 $$\begin{aligned} r(t) = -\left( \lambda \, e_r(t)^2 + \beta \, u(t)^2 \right) \end{aligned}$$

**Fig. 3:**
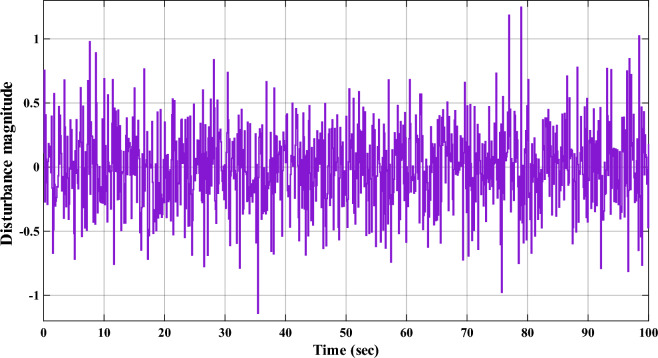
Uncertainties considered due to multiple influencing factors.

In (8), $\lambda$ and $\beta$ are error penalty factor and action penalty factor, respectively. Value of $\lambda$ is considered as 1 whereas the value of $\beta$ as 0.01. Controlled signal, *u*(*t*), represents the output of TD3 RL agent. Numerous external factors, such as operating temperature, unbalanced power supply, ventilation system, environmental conditions^34,35^, etc. impact the efficacy of the DC motor. Hence, robustness of the proposed adaptive controller is tested by utilizing the uncertainties block in overall speed regulation strategy. This block adds random noise into overall speed regulation system as depicted in Fig. 3. This uncertainties are added by utilizing band-limited white noise block which has noise power of 0.01, a sample time of 0.1 seconds, and a seed value of 23341. It is added to controller’s output which is taken as input to the DC motor model as depicted in Fig. 1.

**Table 3:** TD3 hyperparameters.

S. N.	Hyperparameters	Value
1	Mini batch size	128
2	Gradient decay factor	0.9
3	Policy update frequency	2
4	Actor-network’s learning rate	$10^{-2}$
5	Experience buffer length	$10^6$
6	Critic-network’s learning rate	$10^{-2}$
7	Target smooth factor	0.005
8	Target update frequency	2
9	Discount factor	0.99
10	TD3 activation function	ReLU
11	Gradient threshold method	*l*2 norm

### Comparative analysis against benchmark controllers

TD3 RL-based adaptive speed controller’s performance is evaluated against benchmark controllers from the literature. Key controllers techniques used in comparative analysis include Klein et al.^57^, Fertik and Sharpe^58^, McMillan^59^, and Edgar et al.^60^. These benchmark controllers utilized FOPDT model of system in order to design desired controller for any specific purpose. FOPDT model’s generalized expression is mentioned as

9 $$\begin{aligned} D_{fopdt} (s) = \frac{K_m}{sT_m+1}e^{-s\tau _d} \end{aligned}$$

where $K_m$ is gain, $T_m$ is time-constant, and $\tau _d$ is delay time of approximated process. The FOPDT model of any TF based model can be obtained by utilizing different approximation techniques such as Maclaurin expansion^61^, Skogestad half rule method^62^, relay feedback^63^, and the process reaction curve method^64^, etc. This work utilized the process reaction curve method in order to obtain the approximated FOPDT model of the DC motor’s TF system as mentioned in (10).

10 $$\begin{aligned} D_{fopdt} (s) = \frac{9.1731}{4.2074s+1}e^{-0.1321s} \end{aligned}$$

**Fig. 4:**
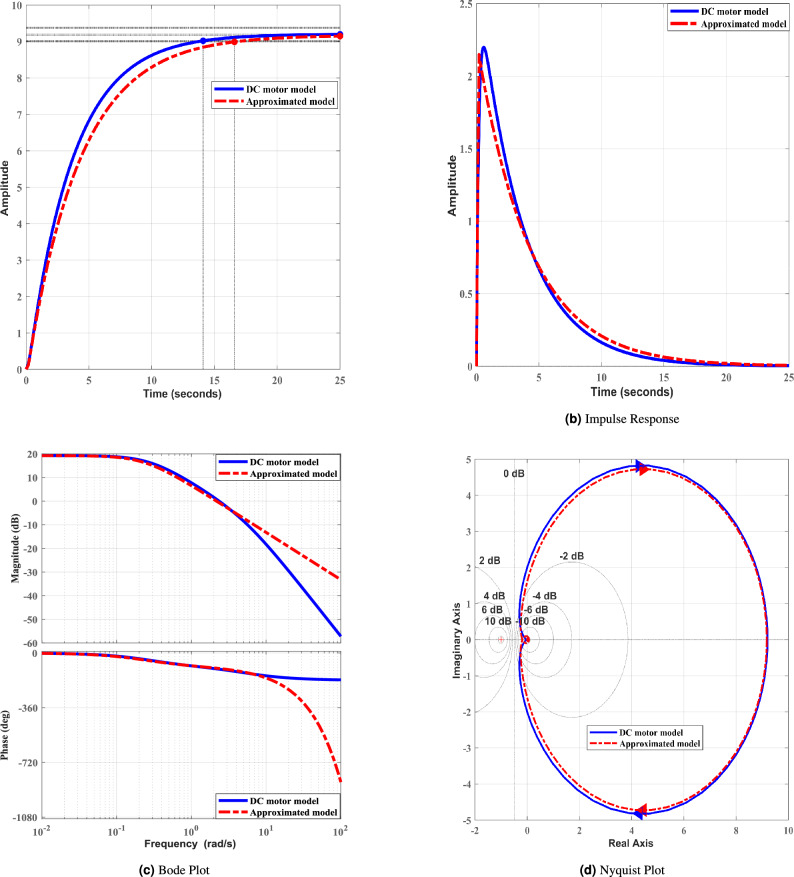
Comparison between the DC motor model and its approximated FOPDT model. (**a**–**d**) represent step response, impulse response, Bode plot, and Nyquist plot, respectively.

Figure 4 presents a comparative analysis of DC motor TF model and its approximated FOPDT model. The step and impulse responses of DC motor TF model and its FOPDT model are given in Fig. 4a and b, respectively. Both responses (Fig. 4a and b) illustrate that the approximated FOPDT model closely follows the original DC motor system’s transient behavior with minimal deviation. Additionally, the Bode plot (given in Fig. 4c) highlights the frequency response characteristics, where the magnitude and phase plots indicate a reasonable approximation of the original system. Finally, the Nyquist plot, as presented in Fig. 4d, demonstrates the stability and frequency domain similarity of the approximated model to the actual DC motor. These results validate that the obtained approximated FOPDT model is maintaining essential system behavior of DC motor system dynamics, making it suitable for further utilization in this work in order to design and implementation of benchmark controller techniques.

This work utilized proportional and integral (PI) controller for speed regulation of DC motor system. Standard PI controller’s structure is given as:

11 $$\begin{aligned} C_{speed}(s) = K_p ( 1 + \frac{1}{sT_i}) \end{aligned}$$

In (11), $K_p$ and $T_i$ are controller’s gains which can be derived utilizing benchmark controller^57–60^ techniques as indicated in Table 4.

**Table 4:** Formulae for controller parameters for different techniques.

S. N.	Controller technique	$K_p$	$T_i$
1	Klein et al.	$\frac{0.28 T_m}{K_m (\tau _d + 0.1 T_m)}$	$0.53 T_m$
2	Fertik and Sharpe	$\frac{0.56}{K_m}$	$0.65 T_m$
3	McMillan	$\frac{K_m}{3}$	$\tau _d$
4	Edgar et al.	$\frac{0.4}{K_m}$	$0.5 T_m$

Values of $K_m$, $\tau _d$, and $T_m$ are obtained as 9.1731, 0.1321, and 4.2074, respectively by comparing (9) and (10). These values are put in Table 4 for calculating the controller gains. After putting these values, Table 5 is obtained.

**Table 5:** Values for controller parameters for different techniques.

S.N.	Controller technique	$K_p$	$T_i$
1	Klein et al.	0.2323	2.23
2	Fertik and Sharpe	0.061	2.735
3	McMillan	3.0577	0.1321
4	Edgar et al.	0.0436	0.06605

## Simulation results and discussion

Response of proposed TD3 RL based adaptive controller is observed along with of all benchmark controllers for two different test cases for tracking desired speed while considering various uncertainties from environment into account. First test case is considered for 45 kmph speed which remains constant for overall simulation time. Later, the desired speed is considered as variable for evaluating controller’s adaptability, transition performance, and stability in dynamic operating conditions for second test case.

### Test case 1: Desired speed remains constant

**Fig. 5:**
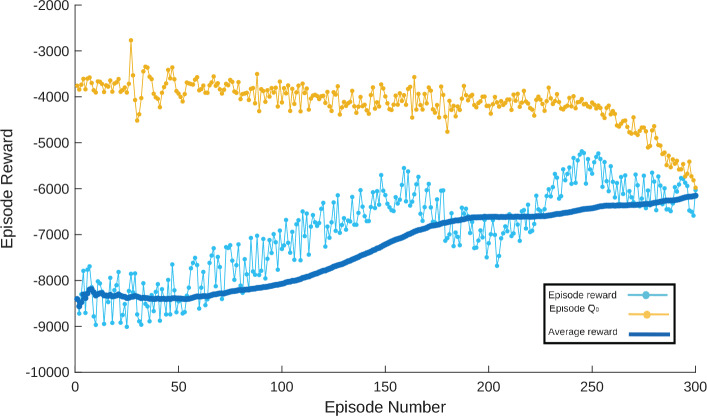
Convergence curves of TD3RL agent’s training.

The training convergence profile of the TD3RL agent is illustrated in Fig. 5. The training graph demonstrated the stable learning process over 300 episodes. The episode reward demonstrating consistent improvement until convergence which confirm the successful learning and effective control policy.

**Fig. 6:**
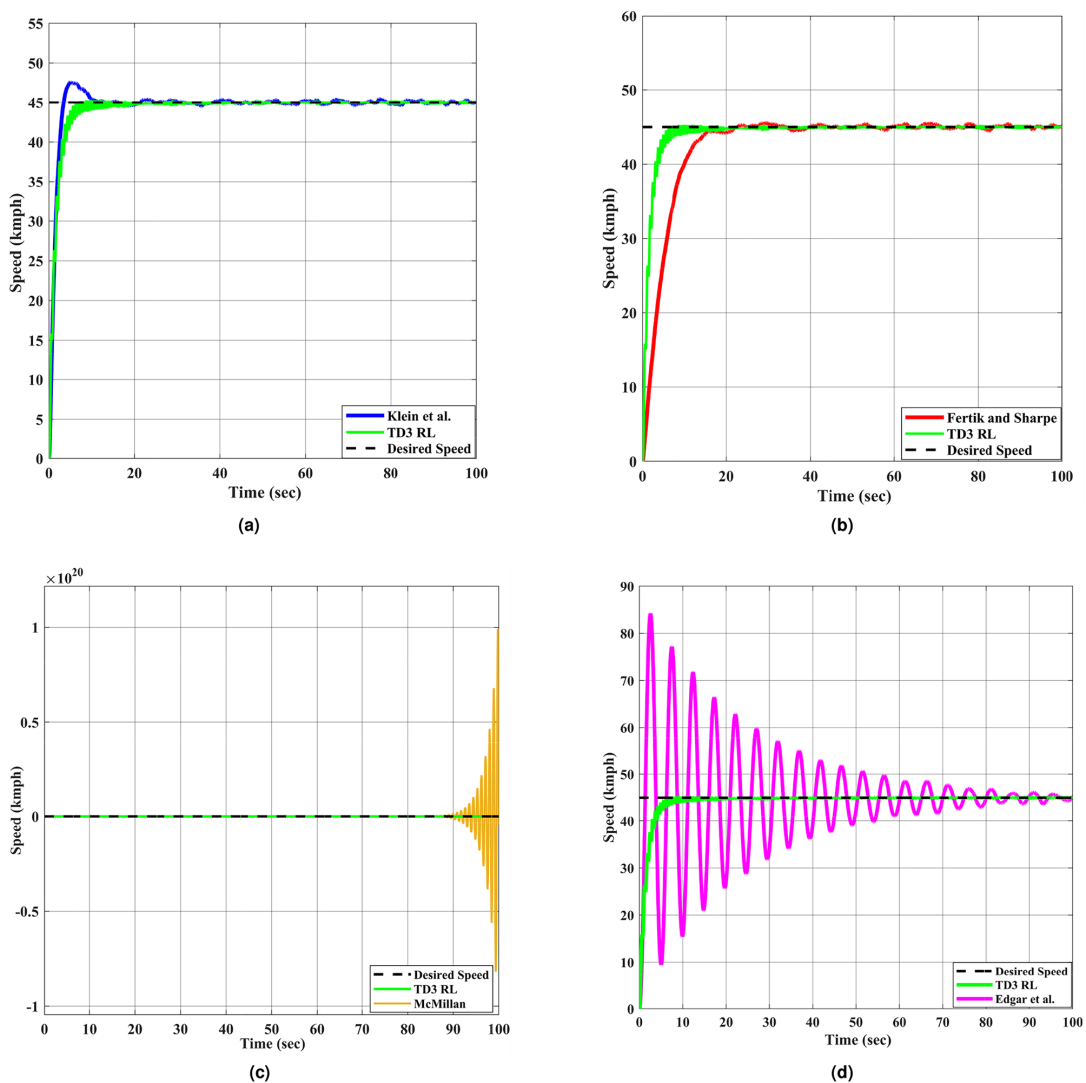
The DC motor response corresponding to constant desired speed. (**a**–**d**) represent different approaches compared with TD3 RL based adaptive controller.

Responses of all controllers which are mentioned in Table 5 are plotted in Fig. 6 along with TD3 RL based controller for tracking constant desired speed which is 45 kmph. Values of various types of error indices, including integral of squared error (ISE), integral of time-weighted absolute error (ITAE), integral of absolute error (IAE), integral of time-weighted squared error (ITSE), and their respective time-weighted variants like integral of time-squared absolute error (IT$^{2}$AE) and integral of time-squared squared error (IT$^{2}$SE), are utilized to evaluate the effectiveness of all controllers. Formulae for these key error indices are mentioned in Table 6, where $e_r(t)$ is error signal and *T* is overall time.

**Table 6:** Key performance indices and their mathematical formulations.

S.N.	Performance index	Formula
1	ISE	$\int _0^T e_r^2(t) \, dt$
2	IAE	$\int _0^T |e_r(t)| \, dt$
3	ITAE	$\int _0^T t |e_r(t)| \, dt$
4	ITSE	$\int _0^T t e_r^2(t) \, dt$
5	IT$^2$AE	$\int _0^T t^2 |e_r(t)| \, dt$
6	IT$^2$SE	$\int _0^T t^2 e_r^2(t) \, dt$

**Table 7:** Key error indices for various controller techniques for constant desired speed.

S.N.	Method	ISE	IAE	ITAE	ITSE	IT^2^AE	IT^2^SE
1	Klein et al.	$1.7{\times }10^3$	$2.1{\times }10^2$	$6.7{\times }10^4$	$1.7{\times }10^4$	$4.3{\times }10^7$	$1.0{\times }10^7$
2	Fertik & Sharpe	$5.7{\times }10^3$	$4.2{\times }10^2$	$1.0{\times }10^5$	$4.7{\times }10^4$	$6.5{\times }10^7$	$2.2{\times }10^7$
3	McMillan	$6.0{\times }10^{301}$	$1.6{\times }10^{154}$	$1.5{\times }10^{154}$	$5.7{\times }10^{304}$	$1.4{\times }10^{157}$	$5.3{\times }10^{307}$
4	Edgar et al.	$1.2{\times }10^4$	$1.0{\times }10^3$	$2.0{\times }10^5$	$2.6{\times }10^5$	$1.2{\times }10^8$	$8.0{\times }10^7$
5	TD3 RL	$\bf {2.9{\times }10^1}$	$\bf{9.9{\times }10^1}$	$\bf{4.1{\times }10^4}$	$\bf{6.0{\times }10^3}$	$\bf{2.6{\times }10^7}$	$\bf{3.7{\times }10^6}$

The results from Fig. 6 indicate that the TD3 RL based adaptive controller exhibits superior tracking capability for DC motor model with fast convergence and minimal overshoot to the desired speed. In contrast, the Klein et al. and Fertik and Sharpe controllers demonstrate a slower response, with noticeable overshoot and settling time. The McMillan controller suffers from extreme instability, as reflected in its high oscillatory response. Similarly, the Edgar et al. controller exhibits excessive oscillations before stabilizing, making it inadequate for precise speed regulation.

The quantitative analysis in terms of error indices is presented in Table 7. This table further confirms effectiveness of the TD3 RL controller against rest of benchmark controller techniques. It achieves the lowest ISE value of 28.6, a significant improvement over Klein et al. (i.e. 1724), Fertik and Sharpe (i.e. 5713), and Edgar et al. (i.e. 1.174e+04). McMillan controller’s extremely high error values indicate an unstable response across all metrics, highlighting its unsuitability for precise speed control. The IAE value of 99.31 for TD3 RL is the lowest, reflecting better sustained error minimization. Similarly, for ITAE and ITSE metrics, TD3 RL achieves 4.14e+04 and 5977, significantly outperforming the others. Additionally, the significant reductions are also observed for the higher-order error indices (IT$^2$AE and IT$^2$SE) for proposed adaptive controller. That further confirms the robustness and efficacy of TD3 RL based speed controller for DC motor system.

### Test case 2: Desired speed is considered as variable

**Fig. 7:**
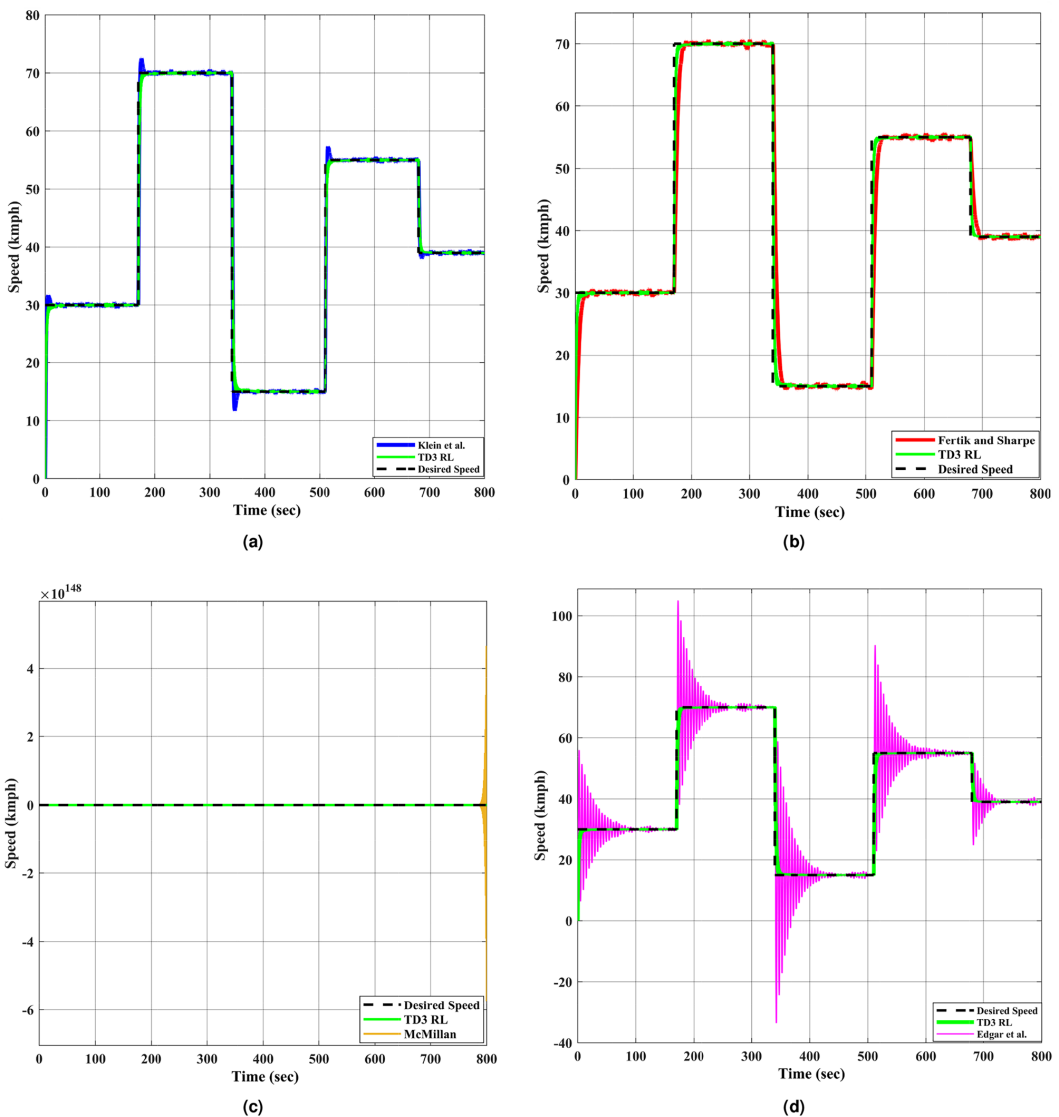
The DC motor response corresponding to variable desired speed. (**a**–**d**) represent different approaches compared with TD3 RL based adaptive controller.

In real world application scenarios, the speed of DC motor rarely remains constant for entire operation period. Designed speed controller may perform well for constant speed but it might fail to adjust the desired speed in dynamic operating conditions. That’s why it become necessary to test the performance of all controllers for variable desired speeds in order to evaluate their efficacy while ensuring smooth transitions between speeds. This test case is considered to verify the stability, robustness, and tracking accuracy of all DC motor speed controllers. Responses of all controllers, which are tested earlier, are plotted in Fig. 7 for tracking variable desired speed.

As observed from Fig. 7, the TD3 RL controller successfully tracks the sharp changes in desired speed with minimal steady-state error and rapid adaptation. In contrast, the Klein et al. and the Fertik and Sharpe controllers exhibit slower transient responses, leading to noticeable tracking delays in desired speed for DC motor system. The McMillan controller again demonstrates excessive oscillations, failing to provide stable control over varying speed demands. Similar excessive oscillations are also observed for the Edgar et al. controller before settling to desired speed. The unstable behavior observed in both the Edgar et al. and McMillan controller techniques highlights their limitations in handling dynamic speed variations effectively.

**Table 8:** Key error indices for various controller techniques for variable desired speed.

S.N.	Method	ISE	IAE	ITAE	ITSE	IT${^2}$AE	IT${^2}$SE
1	Klein et al.	$6.2{\times }10^3$	$3.7{\times }10^2$	$1.3{\times }10^5$	$1.9{\times }10^6$	$6.4{\times }10^7$	$7.8{\times }10^8$
2	Fertik & Sharpe	$2.1{\times }10^4$	$1.0{\times }10^3$	$3.4{\times }10^5$	$6.5{\times }10^6$	$1.6{\times }10^8$	$2.7{\times }10^9$
3	McMillan	$1.0{\times }10^{257}$	$6.8{\times }10^{128}$	$5.4{\times }10^{131}$	$5.4{\times }10^{131}$	$4.3{\times }10^{134}$	$6.6{\times }10^{262}$
4	Edgar et al.	$1.2{\times }10^4$	$1.0{\times }10^3$	$2.0{\times }10^5$	$2.6{\times }10^5$	$1.2{\times }10^8$	$8.0{\times }10^7$
5	TD3 RL	$\bf{6.8{\times }10^1}$	$\bf{1.2{\times }10^2}$	$\bf{4.2{\times }10^4}$	$\bf{2.2{\times }10^4}$	$\bf{2.1{\times }10^7}$	$\bf{9.3{\times }10^6}$

The comparison among error indices, presented in Table 8, further validate the superiority of the TD3 RL controller compared to the rest of benchmark controller techniques. It maintains the lowest ISE, IAE, ITAE, and ITSE values, indicating enhanced tracking accuracy and adaptability. The ISE value for TD3 RL based controller is 67.58. It is far better than next best value of 6219 (Klein et al.), indicating exceptional tracking precision. McMillan again exhibits an unstable response with excessively high values for all types of performance indices. The IAE value of 116.2 is the lowest for proposed controller, showing consistent error minimization. Further, in the ITAE and ITSE metrics, TD3 RL records 4.243e+04 and 2.179e+04, which are far better than rest of control strategies. The significant reduction is again observed in higher-order error indices (IT²AE and IT²SE) which further supports that the TD3 RL based adaptive controller’s outperformed to minimize fluctuations and energy consumption during speed transitions. The improved performance under dynamic speed variations demonstrates the capability of the TD3 RL-based controller to handle real-world operating scenarios for DC motor system more effectively than benchmark controller techniques.

### Analysis of control effort and performance trade-offs

The evaluation of integrated absolute control effort for each controller technique is critical parameter to quantifies the total control energy expended by any controller. It is also practical consideration for measuring the system efficiency and overall energy consumption. The control effort $J_{effort}$ is defined as:

12 $$\begin{aligned} {J_{effort} = \int _{0}^{T} |u(t)| dt} \end{aligned}$$

where *u*(*t*) is the control signal and *T* is the simulation time.

Both constant and variable desired speed cases are taken into consideration for calculation of the $J_{effort}$. The results are demonstrated in Table 9 reveal a critical performance trade-off.

**Table 9:** Control-effort metric for both test cases.

S.N.	Method	Test case-1	Test case-2
1	Klein et al.	502.2	3679
2	Fertik and Sharpe	**483.5**	**3644**
3	McMillan	$7.805 \times 10^{20}$	$4.524 \times 10^{149}$
4	Edgar et al.	616	4176
5	TD3 RL	533.3	3832

The TD3 RL controller achieves a control effort of 533.3 and 3832 in test case 1 and 2, respectively. It’s performance is highly competitive and lies within a narrow margin of the best-performing classical controllers by this metric. This marginal difference in energy consumption must be acknowledge in the context of overall performance.

While the Fertik and Sharpe method shows a slightly lower control effort, this minor advantage comes at a significant cost to desired speed tracking precision, as interpreted by its higher error indices reported in Tables 7 and 8. Whereas, the proposed TD3RL method presents a superior balance, delivering near-optimal control efforts while simultaneously achieving the lowest tracking errors indices. It avoids the undesirable extreme control effort of the McMillan method and also provides a more stable solution than the Edgar et al. technique. Therefore, the TD3 RL controller emerges as the most balanced performing method after considering trade-off between energy consumption and desired speed tracking accuracy.

### Impact of setpoint filter on response of TD3RL agent

**Fig. 8:**
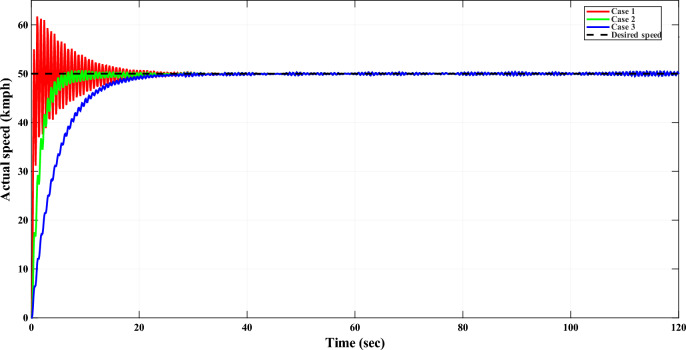
Response due to various setpoint filter on DC motor system for constant desired speed.

**Fig. 9:**
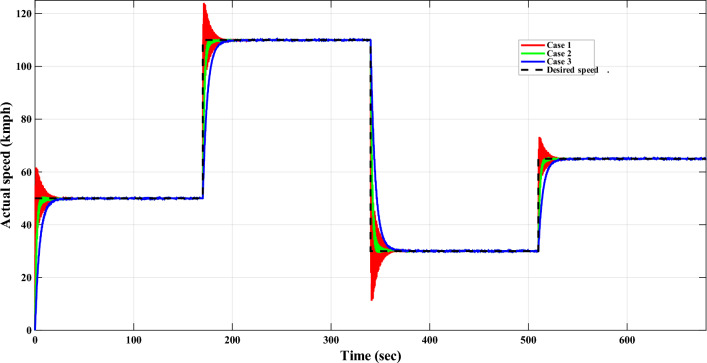
Response due to various setpoint filter on DC motor system for variable desired speed.

The setpoint filter plays critical role in determining how aggressively the desired speed trajectory is followed by the TD3 agent. Three test cases have been performed to evaluate the impact of $\alpha _f$ in which it’s value is taken as 0.5, 1.5, and 4.5, respectively. The response from all these test cases are plotted in Figs. 8 and 9 for constant and variable desired speed, respectively.

The $\alpha _f$ of 1.5 is identified as the optimal value. It provides a fast response with minimal overshoot and, most critically. Comparatively, it eliminates the undesired oscillations observed as with the 0.5 value of $\alpha _f$ , while avoiding the excessive overshoot of the 4.5 value.

## Conclusion and future work

This study proposed the design and implementation of TD3 RL based adaptive speed controller for the DC motor model while considering various types of uncertainties in order to test it’s performance in dynamic operating scenario. Both qualitative and quantitative results indicate that TD3 RL based controller consistently outperformed rest of benchmark controller techniques across all error indices for both constant and variable desired speed test cases, indicating its higher adaptability, efficacy, and robustness. Proposed adaptive controller exhibits least values for both IAE and ISE, for both test cases, which indicates exceptional tracking precision and error minimization qualities. Further, the lowest values for higher-order error indices (IT$^2$AE and IT$^2$SE) suggest that the proposed TD3 RL based adaptive speed controller maintains performance despite uncertainties in dynamic environment for both test cases. Thus, this study highlighted the potential of RL in the DC motor speed regulation in efficient manner. The presented research work is achieved via software-based validation in MATLAB Simulink environment. Future work will focus on bridging this gap through hardware-in-loop (HIL) validation on a real DC motor setup under parameter variation and a comparative analysis with other RL algorithms, such as SAC, PPO, and A3C, to detailed comparative analysis. Multiple safety protocol should be followed for transition from simulation to practice such as defining ramp rate for desired speed variation and setting up HIL setup for continuous feedback on TD3 agent’s training. Additionally, a supervisory safety layer should be deployed for monitoring for fault conditions such as over-current, excessive speed deviation, or abnormal winding temperatures. Emergency-stop mechanisms would be critical in order to override the RL agent’s commands for prevention of damage to the DC motor system in case of any contingency occurs.

The performance of TD3RL algorithm is sensitivity to hyperparameter selection, particularly to replay buffer, discount factor and total number of episodes for training period. Hence, the values of hyper-parameters used in this research work may not be generalize to other industrial systems without extensive retraining of agent. Additionally, the memory requirements grow substantially with replay buffer size and complexity of the operating system. The implementation of TD3RL algorithm framework to complex industrial objectives is future scope of this work. The TD3RL can be utilized in autonomous drone navigation as it has robust capability for handling of aerodynamic disturbances and providing smooth operating control. Similarly, in load frequency control for smart grids, the trained TD3 RL can enables continuous adjustments to maintain grid frequency while taking fluctuating power demands and non-linear grid dynamics into the account. Additionally, TD3 can be utilized for the speed control of electric vehicles by efficiently handling external driving uncertainties like road gradient, temperature, speed limit, rate of traffic flow, etc.

## Data Availability

All data generated or analyzed during this study are included in this article.
